# Prevalence of human papillomavirus infection in women in Benin, West Africa

**DOI:** 10.1186/1743-422X-8-514

**Published:** 2011-11-10

**Authors:** Franca Piras, Michela Piga, Antonella De Montis, Ahissou RF Zannou, Luigi Minerba, Maria T Perra, Daniela Murtas, Manuela Atzori, Marco Pittau, Cristina Maxia, Paola Sirigu

**Affiliations:** 1 Department of Cytomorphology, University of Cagliari, Cagliari, Italy; 2 Department of Pathology, SS. Trinità Hospital, Cagliari, Italy; 3 Research Laboratories, bcs Biotech S.p.A., Cagliari, Italy; 4 Ministère de la Santé, Cotonou, Bénin; 5 Department of Public Health, University of Cagliari, Cagliari, Italy

**Keywords:** human papillomavirus, cervical cancer, Benin, Pap test, prevention

## Abstract

**Background:**

Cervical cancer ranks as the first most frequent cancer among women in Benin. The major cause of cervical cancer now recognized is persistent infection of Human Papillomavirus (HPV). In Benin there is a lack of screening programs for prevention of cervical cancer and little information exists regarding HPV genotype distribution.

**Methods:**

Cervical cells from 725 women were examined for the presence of viral DNA by means of a polymerase chain reaction (PCR) multiplex-based assay with the amplification of a fragment of L1 region and of E6/E7 region of the HPV genome, and of abnormal cytology by Papanicolaou method. The association between HPV status and Pap test reports was evaluated. Socio-demographic and reproductive characteristics were also related.

**Results:**

A total of 18 different HPV types were identified, with a prevalence of 33.2% overall, and 52% and 26.7% among women with and without cervical lesions, respectively. Multiple HPV infections were observed in 40.2% of HPV-infected women. In the HPV-testing group, the odds ratio for the detection of abnormal cytology was 2.98 (95% CI, 1.83-4.84) for HPV positive in comparison to HPV negative women. High risk types were involved in 88% of infections, most notably HPV-59, HPV-35, HPV-16, HPV-18, HPV-58 and HPV-45. In multiple infections of women with cytological abnormalities HPV-45 predominated.

**Conclusions:**

This study provides the first estimates of the prevalence of HPV and type-specific distribution among women from Benin and demonstrates that the epidemiology of HPV infection in Benin is different from that of other world regions. Specific area vaccinations may be needed to prevent cervical cancer and the other HPV-related diseases.

## Background

Benin has a population of 2.22 million women aged 15 years and older who are at risk of developing cervical cancer. Cervical cancer ranks as the first most frequent cancer among women in Benin, and the second most frequent cancer among women between 15 and 44 years of age. Current estimates indicate that every year 925 women are diagnosed with cervical cancer and 616 die from the disease [1].

There is an annual global incidence of nearly 500 000 new cervical cancer cases, with 274 000 deaths per year [2]. More than 80% of these cases occur in developing countries, and this percentage is expected to increase to nearly 90% by the year 2020 [3].

In developing countries, such as Benin, there is a lack of effective screening programs for prevention of cervical cancer. In these countries, in fact, no clinically significant reduction in the incidence of cervical cancer has occurred during the past three decades [2,4]. In developed countries, by contrast, there has been a major decline in cervical-cancer mortality after the introduction of large-scale cytological testing [2]. The introduction of the Pap test into general screening programs in the United States has reduced the incidence of advanced forms of cervical cancer by 75% over the past few decades [5,6].

The major cause of cervical cancer now recognized is persistent infection of HPV [7]. HPV exists in a surprisingly large number of different types: to date > 100 have been identified and it has been anticipated that almost 200 may exist [8]. Cervical infections by approximately 15 cancer-associated (carcinogenic or high-risk) HPV genotypes (HR-HPV) cause virtually all cervical cancers and their immediate precursors worldwide [9].

The distribution of HPV types varies greatly across populations and HPV surveys have been performed in different geographical regions in order to apply appropriate vaccine strategies [10]. However, little information exists regarding HPV genotype distribution in women from developing countries at high incidence of cervical cancer and, concerning Benin, data are not yet available on the HPV burden in the general population [1].

In light of these considerations, the aim of this study was to examine, through an observational study, the HPV genotype distribution and the cervical cell cytology among women from Benin by evaluating genital swabs collected from 725 women undergoing voluntary screening. In addition, socio-demographic and reproductive characteristics were related with HPV and cytological results.

## Methods

### Study Population and Sample Collection

From January to September 2009 a total of 725 genital swabs were collected from women aged from 15 to 70 years undergoing voluntary screening at Saint Luc Hospital of Cotonou and Porto-Novo (south Benin), at the Hospital of Abomey, at the St. John Catholic Hospital of Parakou and of Lokossa (south-central Benin), at the Hôpital de L'Ordre de Malte of Djougou, at Saint Jean de Dieu Fatebenefratelli of Tanguetà, and Natitingou in Atakora Department (north-west Benin), and at the main maternity hospital (HOMEL, Hôpital Mère-Enfant de la Lagune). The study protocol was approved by the local research ethic committee, and informed consent was obtained from all subjects according to the World Medical Association Declaration of Helsinki. All participants were interviewed using a structured questionnaire with respect to sociodemographic and reproductive characteristics such as age, age at first sexual activity, number of pregnancies, age at first menstruation, and number of abortions. They were also instructed about the causes of cervical cancer, signs and symptoms, prevention, early detection, and treatment. Results of HPV and cytological testing were delivered to the health centres after testing, and those with positive tests were suggested for colposcopy, biopsy, and adequate treatments.

Cervical smears, suspended in ThinPrep preservative solution, were processed for HPV types at Research Laboratories, bcs Biotech S.p.A., Cagliari, Italy, on the basis of the manufacturer's instructions. In the cytological testing, cervical cells were collected with the use of the Ayre's spatula (esocervix) and cytobrushes (endocervix). The smears were processed in the Cytomorphology Department laboratories of Cagliari University, Italy, and independently evaluated by two anatomopathologists, according to the 2001 Bethesda system [11,12].

### Training

Screening was performed by doctors and auxiliary nurse-midwives who were trained in a preventive course using International Agency for Research on Cancer (IARC) manuals for the collection of cervical cells for HPV and cytological testing.

### Cytology

All the women were screened for the presence of abnormal cervical cytology with conventional Pap smears. Interpretation of the cytological specimens was reported according to the Bethesda System 2001, adopted in May 2001 and including revision statements of specimen adequacy, general categorization, interpretation and result of epithelial cell abnormalities as follows: "atypical squamous cells" (ASC) divided into qualifiers of 1) ASC of "undetermined significance" (ASC-US), and 2) "cannot exclude high-grade squamous intraepithelial lesion" (ASC-H); low-grade squamous intraepithelial lesion (LSIL); high-grade squamous intraepithelial lesion (HSIL); squamous cell carcinoma; atypical glandular cells (AGC), with attempts to identify whether the origin of the cells was endometrial, endocervical or unqualified; endocervical adenocarcinoma *in situ *and "AGC favour neoplastic". Any abnormalities detected were dealt with in compliance with guidelines updated by the Italian Association of Colposcopy and Cervical/Vaginal Pathology [13-17].

### HPV DNA Typing

HPV DNA typing was performed by the Biochip array genotyping test based on four major processes that include specimen preparation, using the ProDect^® ^HPV Extraction kit (bcs BIOTECH), multiplex PCR amplification of target DNA, hybridization of amplified products to oligonucleotide probes, and finally detection of the amplified products by colourimetric determination, using the ProDect^® ^Chip HPV Typing kit (bcs BIOTECH). The test system uses L1 viral region (143 bp) as target to detect and type 14 viruses with a high-medium oncogenic risk (HPV-16, HPV-18, HPV-31, HPV-33, HPV-35, HPV-39, HPV-45, HPV-51, HPV-52, HPV-56, HPV-58, HPV-59, HPV-68, HPV-73), and 5 viruses with a low-oncogenic risk (HPV-6, HPV-11, HPV-42, HPV-43, HPV-44). Moreover, to increase the rate and complete the HPV detection, this method amplifies the E6 and E7 ORF (233-268 bp) when the L1 system gives negative results [18-22].

DNA extraction was carried out with the ProDect^® ^HPV Extraction kit (bcs BIOTECH, Cagliari, Italy) as follows: 3 ml of ThinPrep cytological suspension were centrifuged at 2000 rpm for 20'; cell pellets were resuspended in a lysis buffer and digested with Proteinase K. The total DNA extracted was concentrated with an alcoholic precipitation and resuspended with 30 μl of Tris-EDTA buffer.

DNA amplification was performed by multiplex PCR with the ProDect^® ^Chip HPV Typing kit (bcs BIOTECH, Cagliari, Italy): each 50 μl reaction consisted of working mix containing MgCl2, KCl, TRIS-HCl, Taq DNA polymerase, dNTPs, and biotinylated GP5+/GP6+, pU-1M/pU-2R primers and β-globin primers together with 5 μl of DNA sample. The PCR was programmed as follows: 94°C for 2 min, and 5 cycles of 94°C for 30 sec, 50°C for 1 min, 72°C for 30 sec and 40 cycles of 94°C for 30 sec, 45°C for 1 min, 72°C for 30 sec and finally, at 72°C for 10 min before cooling it indefinitely at 4°C.

Hybridization to the oligonucleotide probe: denaturing solution was added to the PCR product; all washes and hybridization steps were undertaken in a 96-microplate wells in which the specific probes for 19 HPV genotypes, E6/E7 sequences, and β-globin reference were pre-spotted [23]. The system also included a hybridisation control probe to monitor the phase of detection on the chip, and a PCR control probe to check the phase of amplification and extraction.

Colourimetric determination: the colour change reaction was from Streptavidin-phosphatase mediated precipitation of working substrate.

The Biochip reading and pattern interpretation were performed through an automated reader and specifically designated software (ProDect^® ^Bcs Biochip Reader - bcs BIOTECH, Cagliari, Italy). Based on the microbiological profile of the sample, different patterns appeared on the chip, each corresponding to the type of pathogen potentially present in the starting sample.

To confirm the results of Biochip typing method, the PCR product was also sequenced using the Big Dye Terminator Cycle Sequencing Kit (Applied Biosystems).

### Statistical Analysis

Statistical analysis was performed by Statistical Package for the Social Sciences 15.0 software. All variables were expressed as absolute and relative frequencies.

The association between HPV status and Pap test reports was evaluated by calculating crude odds ratio (OR) by logistic regression analysis with a 95% confidence interval (CI) and by Fisher's exact test. The association between HPV or Pap test status and clinical-gynaecological variables, such as number of pregnancies, number of abortions, menopausal status, and pregnancy status, was assessed through the χ^2 ^test or Fisher's exact test (significant *P *value < 0.05). Mann-Whitney and Kruskal-Wallis tests were used to analyze differences between age at diagnoses, age at first sexual activity, age at menarche, age at first childbirth and HPV or Pap test status for all women.

To deal with missing data, a multiple imputation model with fully conditional specification was used.

## Results

Of the 725 women who came to the clinic study 427 had valid HPV tests, whereas 298 have no adequate sample to HPV testing.

Viral DNA was identified in 142 of the 427 samples (33.2%); 85 (59.8%) samples had single infection and 57 (40.2%) had multiple infection. Among HPV-positive samples, 125 (88%) had high-risk HPV infection (Table 1).

**Table 1:** HPV type distribution (%) in single and multiple infections in 142 women in Benin, West Africa

HPV genotype	Total infections (n = 142) n (%)	Single infections (n = 85) n (%)	Multiple infections (n = 57) n (%)	***P***^**a**^
**High-risk**				
**HPV-59**	35 (24.65)	20 (23.53)	15 (26.32)	0.705
**HPV-35**	32 (22.54)	11 (12.94)	21 (36.84)	0.001
**HPV-16**	25 (17.61)	10 (11.76)	15 (26.32)	0.026
**HPV-18**	21 (14.79)	8 (9.41)	13 (22.81)	0.028
**HPV-58**	19 (13.38)	9 (10.59)	10 (17.54)	0.233
**HPV-45**	14 (9.86)	3 (3.53)	11 (19.30)	0.002
**HPV-56**	12 (8.45)	9 (10.59)	3 (5.26)	0.263
**HPV-73**	7 (4.93)	3 (3.53)	4 (7.02)	0.347
**HPV-33**	6 (4.23)	0 (0.00)	6 (10.53)	0.002
**HPV-51**	5 (3.52)	0 (0.00)	5 (8.77)	0.005
**HPV-31**	5 (3.52)	1 (1.18)	4 (7.02)	0.064
**HPV-52**	4 (2.82)	2 (2.35)	2 (3.51)	0.683
**HPV-39**	1 (0.70)	1 (1.18)	0 (0.00)	0.411
**Low-risk**				
**HPV-42**	11 (7.75)	7 (8.24)	4 (7.02)	0.790
**HPV-6**	10 (7.04)	0 (0.00)	10 (17.54)	0.000
**HPV-11**	10 (7.04)	0 (0.00)	10 (17.54)	0.000
**HPV-43**	6 (4.23)	0 (0.00)	6 (10.53)	0.002
**HPV-44**	1 (0.70)	1 (1.18)	0 (0.00)	0.411

^a^Single infection versus multiple infection, Z-test or Fisher' s exact test.

HPV-59 and HPV-35 were the most commonly detected types, followed by other high-risk genotypes such as HPV-16 (17.6%), HPV-18 (14.8%), HPV-58 (13.4%), HPV-45 (9.9%), HPV-56 (8.4%), and low risk genotypes such as HPV-42 (7.7%), HPV-6 and -11 (7%) in women with both normal and abnormal cytology. HPV-59 was detected both in single and multiple infections, whereas other HR-HPV types (i.e., HPV-35, HPV-16, HPV-18, and HPV-45) were more frequently identified in multiple infections (Table 1).

Among the 427 women who had a HPV testing, 354 had both Pap test and HPV testing (Table 2). Among the 354 women HPV DNA was found in 26.7% of 258 women with negative Pap tests and in 52% of 96 abnormal Pap tests. Seventy-two ASC-US, 7 LSILs, 4 HSILs, 7 ASC-H, 6 AGC were found among the 96 women with abnormal Pap test. Among women with both abnormal Pap smear and HPV infection (n = 50), 96% had HR-HPVs. In the HPV-testing group, the odds ratio for the detection of abnormal cytology was 2.98 (95% CI, 1.83-4.84) for HPV positive in comparison to HPV negative women. Fisher's exact test showed a statistically significant correlation between HPV presence and abnormal cytology (*P *< 0.0001).

**Table 2:** Prevalence of HPV types in women in Benin with normal and abnormal cytological results

Pap test and HPV performed samples (n = 354)						
**Normal cytology (n = 258)**				**Abnormal cytology (n = 96)**		

**HPV type**	**Single infection** **n (%)**	**Multiple infection** **n (%)**	**Total** **n (%)**	**Single infection** **n (%)**	**Multiple infection** **n (%)**	**Total** **n (%)**

**HPV +**	45 (17.4)	24 (9.3)	69 (26.7)	28 (29.2)	22 (22.9)	50 (52.1)
**High-risk**						
**HPV-59**	11 (24.4)	11 (45.8)	22 (31.9)	7 (25.0)	5 (22.7)	12 (24.0)
**HPV-56**	6 (13.3)	0 (0.0)	6 (8.7)	3 (10.7)	2 (9.1)	5 (10.0)
**HPV-16**	5 (11.1)	6 (25.0)	11 (15.9)	3 (10.7)	6 (27.3)	9 (18.0)
**HPV-18**	4 (8.9)	4 (16.7)	8 (11.6)	3 (10.7)	5 (22.7)	8 (16.0)
**HPV-35**	3 (6.7)	9 (37.5)	12 (17.4)	5 (17.9)	6 (27.3)	11 (22.0)
**HPV-58**	3 (6.7)	5 (20.8)	8 (11.6)	2 (7.1)	2 (9.1)	4 (8.0)
**HPV-45**	3 (6.7)	1 (4.2)	4 (5.8)	0 (0.0)	8 (36.4)	8 (16.0)
**HPV-73**	1 (2.2)	2 (8.3)	3 (4.3)	2 (7.1)	2 (9.1)	4 (8.0)
**HPV-39**	1 (2.2)	0 (0.0)	1 (1.4)	0 (0.0)	0 (0.0)	0 (0.0)
**HPV-31**	1 (2.2)	1 (4.2)	2 (2.9)	0 (0.0)	3 (13.6)	3 (6.0)
**HPV-33**	0 (0.0)	2 (8.3)	2 (2.9)	0 (0.0)	4 (18.2)	4 (8.0)
**HPV-51**	0 (0.0)	3 (12.5)	3 (4.3)	0 (0.0)	2 (9.1)	2 (4.0)
**HPV-52**	0 (0.0)	1 (4.2)	1 (1.4)	2 (7.7)	1 (4.5)	3 (6.0)
**Low-risk**						
**HPV-42**	6 (13.3)	3 (12.5)	9 (13.0)	1 (3.6)	1 (4.5)	2 (4.0)
**HPV-44**	1 (2.2)	0 (0.0)	1 (1.4)	0 (0.0)	0 (0.0)	0 (0.0)
**HPV-6**	0 (0.0)	6 (25.0)	6 (8.7)	0 (0.0)	4 (18.2)	4 (8.0)
**HPV-11**	0 (0.0)	6 (25.0)	6 (8.7)	0 (0.0)	4 (18.2)	4 (8.0)
**HPV-43**	0 (0.0)	2 (8.3)	2 (2.9)	0 (0.0)	3 (13.6)	3 (6.0)

HPV DNA presented in 46% of ASC-US, in 71.4% of LSILs, in 75% of HSILs, in 57.1% of ASC-H, and in 85.7% of AGC.

Tables 3 and 4 show the genotype HPV distribution in the single and multiple infections of the specific cytological lesions.

**Table 3:** HPV-type distribution in single infection of women with abnormal cytology

HPV-type	No. women	ASC-US	ASC-H	AGC	LSIL	HSIL
**HPV-59**	7	4	1	2	0	0
**HPV-35**	5	4	0	0	0	1
**HPV-56**	3	3	0	0	0	0
**HPV-16**	3	3	0	0	0	0
**HPV-18**	3	2	0	1	0	0
**HPV-52**	2	0	1	0	0	1
**HPV-73**	2	2	0	0	0	0
**HPV-58**	2	1	0	0	0	1
**HPV-42**	1	1	0	0	0	0

**Table 4:** HPV-type distribution in multiple infections of 22 women with abnormal cytology

No. women	ASCUS	No. women	ASC-H
1	HPV-45, 35, 16, 33, 59, 58, 52	1	HPV-45, 18, 31
1	HPV-45, 16, 6, 11, 43	1	HPV-35, 59
1	HPV-45, 35, 31, 43		
1	HPV-59, 51, 56, 73		**AGC**
			
1	HPV-45, 33, 43	1	HPV-18, 31
1	HPV-45, 56	1	HPV-16, 33
1	HPV-45, 58		
1	HPV-45, 16		**LSIL**
			
1	HPV-16, 33	1	HPV-59, 51, 42
1	HPV-59, 73	1	HPV-16, 58
3	HPV-35, 18	3	HPV-6, 11

The correlation between the HPV positivity or abnormal cytology and most of clinical-gynaecological characteristics of screened Benin women, such as number of pregnancies, number of abortions, menopausal status, and pregnancy status, assessed by Fisher's exact test, revealed no statistically significant association. Fisher's exact test showed that the abnormal Pap smear appears in 27.9% of women under 36 (mean value) and 18.6% of older ones (*P *= 0.01). Mann-Whitney and Kruskal-Wallis tests showed a significant correlation between age at diagnoses and abnormal cytology (*P *= 0.03). No correlation was found between age at diagnoses and HPV status (*P *= 0.28). These tests have not shown significant correlation between HPV or Pap test status with age at first sexual activity (*P *= 0.8 and *P *= 0.83, respectively), age at menarche (*P *= 0.77 and *P *= 0.92, respectively), and age at first childbirth (*P *= 0.05 and *P *= 0.08, respectively).

## Discussion

For the first time, this study provides information on HPV prevalence and type-specific distribution among women from Benin undergoing voluntary screening. The data show a very high overall prevalence of HPV infection of 33.2% in such women. Moreover, 88% of the Benin women's infections presented high risk HPV types, and 40.1% of the infections involved more than one HPV type (2-7 types).

Most of HPV-DNA positive women analysed in this study were infected with HPV-59, HPV-35, HPV-16, and -18, in decreasing order. The HPV types had different distributions in single and multiple infections. HPV-59 was present with the same proportion in single and multiple infections, while 36.84% of multiple infections presented HPV-35, followed in decreasing order by HPV-16, HPV-18 and HPV-45 (Z-test and Fisher's exact test).

These data on HPV type distribution in women from Benin indicate that the epidemiology of HPV infection in this area is different from other regions of the world and also from those close to Benin such as Nigeria and Burkina Faso. The prevalence of HPV genotypes in cervical cytological samples varies greatly in different geographical regions [24-27]. In fact, both in women with normal cervical cytology and in those with cervical lesions or cancer, HPV-16 is the most common HPV type followed by HPV-18 in Europe, Central and South America, by HPV-52 and HPV-58 in Asia, by HPV-53 and HPV-52 in North America [28,29], HPV-31 and HPV-35 in Nigeria [30]. In Burkina Faso HPV-52, -35 and -58 were detected as the most prevalent types in high risk women, as observed in other African studies [31]. These differences in the pattern of HPV type distribution in countries and regions may be related to different sexual habits and migrations of people [29,32].

The frequency of HPV in cervical cytological samples shows a strong correlation with cervical cancer incidence, but HPV-positivity may be detected in a high rate in women with negative histology or cytology. In this study, HPV DNA was detected in 26.7% of women from Benin without cytological abnormalities and many of whom harboured HPV types with very high progressive potential. These are alarming data since they result very high in comparison to those of a recent meta-analysis that estimated that the worldwide HPV prevalence in the cervix of women with normal cytology is about 10.4% [28]. The data are similar to high values of HPV frequency detected in Nigeria, a region that Benin belongs to. In fact, the population-based HPV surveys coordinated by the IARC reported that Nigeria had the highest prevalence of all HPV types and Europe the lowest, with nearly a 20-fold variation between Nigeria (25.6%) and Spain (1.4%) in women without cytological abnormalities [26,30,33].

The HPV type 16, although with different prevalence rates, is the most common viral type, being present in 12.3%, 18.4%, 21.4% and 25.5% of HPV-positive women without cytological lesions from Sub-Saharan Africa (Nigeria), Asia, South America and Europe, respectively [26]. These data show that not HPV-16 but HPV-59 is the predominant HPV in 258 Benin women without cytological lesions, with a prevalence of 31.9%. Nevertheless, the HPV-35 and HPV-16, also not representing the prevalent types, are present at a very high rate in normal cytological samples (17.4% and 15.9% respectively).

As expected, in women from Benin with abnormal Pap tests HPV DNA was detected with a high rate of 52%. The odds ratio in fact shows that HPV-infected women run a double risk of having a cervical lesion compared to those uninfected. HPV-59 and HPV-35 are the most common HPV types identified in these women with cytological lesions, followed by HPV-16, HPV-18. In multiple infections, HPV-45 was the one mostly detected. These findings are disturbing since previous surveys from nearby geographically regions showed that HPV35 and HPV45 were common in sub-Saharan Africa women with high-grade squamous intraepithelial lesions and squamous cell carcinoma [24-26].

Population-based data for HPV-type distribution is a prerequisite to development of new HPV-screening tests to predict the potential benefits of HPV vaccination and to monitor the impact of vaccination on HPV type replacement, but these data are limited or missing for many world regions [26]. This study for the first time reports the frequency of HPV infection and HPV genotype distribution on a female population from Benin and shows that the most frequent HPV types are HPV-59, HPV-35, HPV-16, HPV-18 and HPV-45 in women with and without cytological lesions. Recently, the Food and Drug Administration approved two HPV vaccine (Gardasil and Cervarix) which are directed against only HPV-6, -11, -16, and HPV-18 of the oncogenic HPV strains [3,34]. However, even though clinical trials of these prophylactic vaccines targeted for HPV-16 and -18 showed a considerable preventive effect for HPV infection and precancerous lesions, the cross-protection among different HPV genotypes remains unsolved in vaccinated women [35,36] and, despite vaccination, other oncogenic strains, more prevalent compared to HPV-16 and 18, as well as HPV-59, HPV-35, and HPV-45 in Benin, would cause infections of different severity. Moreover, the two vaccines have been documented in clinical trials to have a durability of only 5-8 years [34] and long-term protection after this time is unknown. Thus, costly boosters, especially for developing countries, would be necessary to maintain immunity against these HPV genotypes [37,38]. Therefore, the results of this study suggest that to prevent cervical cancer and other HPV-related diseases, the development of other vaccines directed against genotypes more prevalent than HPV-16 and HPV-18, detected in Benin, are needed.

The results of the association between the gynaecological and socio-demographic characteristics and HPV infection or Pap test results are inconsistent except for the relation of abnormal Pap smears to young age, age at first childbirth, and number of spontaneous abortions. Individual sexual habits, difficult to detect in populations, are probably responsible for these data.

## Conclusions

These data can be considered as nationally representative of Benin because the study analysed women from a population living there. A major strength of this study is the fact that it provides the first estimates of the prevalence of HPV among women from the general population in Benin, showing an elevated frequency of genital HPV infection in Benin women. Moreover, the high prevalence of viral types other than vaccine types should be taken into account in developing area-specific vaccines. These data will contribute to elucidating the epidemiology of HPV infection across subpopulations, and it will also be helpful in the implementation of future prevention strategies.

## Abbreviations

AGC: Atypical glandular cells; ASC: Atypical squamous cells; ASC-H: ASC cannot exclude high-grade squamous intraepithelial lesion; ASC-US: ASC of undetermined significance; CI: Confidence interval; HSIL: High-grade squamous intraepithelial lesion; HPV: Human Papillomavirus; HPV LR: Human Papillomavirus of Low-Risk for cancer; HPV HR: Human Papillomavirus of High-Risk for cancer; IARC: International Agency for Research on Cancer; LSIL: Low-grade squamous intraepithelial lesion; OR: Odds ratio; PCR: Polymerase Chain Reaction.

## Competing interests

The authors declare that they have no competing interests.

## Authors' contributions

FP contributed to conception and design, acquisition, analysis and interpretation of data, and drafted the manuscript. MP coordinated the acquisition of the samples and performed the cytological analysis. ADM, MA, and MP performed the molecular procedures and help to draft the manuscript. ARFZ participated in study design and acquisition of the samples. LM performed the statistical analysis. MTP revised the data and contributed with important intellectual content. DM and CM contributed to acquisition and elaboration of data. PS conceived and participated in study design and coordination, and contributed to acquisition of the samples, to draft the manuscript and supervised the work of the research group. All authors read and approved the final manuscript.
